# ChatGPT, Python, and Microsoft Excel

**DOI:** 10.5195/jmla.2025.2065

**Published:** 2025-01-14

**Authors:** Kaique Sbampato, Humberto Arruda, Édison Renato Silva

**Affiliations:** 1 kaique.sbampato@poli.ufrj.br, Production Engineering Program, Universidade Federal do Rio de Janeiro, Rio de Janeiro, Brazil; 2 Humberto.arruda@poli.ufrj.br, Production Engineering Program, Universidade Federal do Rio de Janeiro, Rio de Janeiro, Brazil; 3 edison@poli.ufrj.br, Industrial Engineering Department and Production Engineering Program, Universidade Federal do Rio de Janeiro, Rio de Janeiro, Brazil

## Abstract

**ChatGPT (version 4.0, March 14, 2024).** OpenAI, San Francisco, CA, USA. https://chat.openai.com; free and subscription plans available.

**Python (version 3.12.1, October 2, 2024).** Python Software Foundation, Beaverton, OR, USA. https://www.python.org; free, open-source.

**Microsoft Excel (version 365).** Microsoft Corporation, Redmond, WA, USA. https://www.microsoft.com/excel; proprietary software, subscription-based.

The introduction of ChatGPT to the public marked a transformative shift in productivity and workplace automation, with its long-term impact yet to fully unfold. The medical library sector is no exception: in an age where effective information management and rapid access to relevant data are essential, librarians can leverage Artificial Intelligence (AI) to streamline, expedite, and enhance their daily tasks [1]. These include cataloging and organizing information, information retrieval, supporting clinical decision-making, and managing institutional knowledge, all benefiting from AI's ability to provide an additional layer of review and efficiency.

Librarians are already well-versed in using tools like Microsoft Excel to manage data and organize information. While tools like ChatGPT and Python might initially seem more complex, they are designed to be user-friendly and can greatly boost productivity when incorporated into daily work-flows. Python, now integrated natively into Excel, offers a simple yet powerful coding language that enables medical librarians to automate tasks and perform advanced data analysis without leaving their familiar spreadsheet environment. The combination of Excel and Python can empower librarians to handle larger datasets, automate repetitive tasks, and generate more sophisticated insights with ease.

The integration of AI into professional routines is reshaping expectations across industries. As AI tools become more widespread, there is a growing assumption that leveraging these technologies for data analysis, decision-making, and automation is now a standard part of “doing good work.” In the medical library field, this means that harnessing AI and Python for international collaboration, managing vast amounts of data, and providing rapid, accurate insights is becoming not just an enhancement, but a crucial component for maintaining excellence in research and information management.

## CHATGPT

ChatGPT, a variant of the Generative Pretrained Transformer (GPT) models developed by OpenAI, exemplifies the rapid advancement in natural language processing (NLP) [2]. It leverages deep learning algorithms to understand context and generate coherent, contextually appropriate responses, marking significant milestones in AI development [3]. The first version of GPT was launched in 2018, and the model has since evolved into increasingly sophisticated conversational AI. The latest version, GPT-4, introduced in 2023, enhances these capabilities further, with multimodal abilities to process both text and images. As a result, ChatGPT has quickly become a transformative force across diverse industries, showcasing its adaptability and robust capability in enhancing operational performance and customer interactions [4].

## PYTHON

Python is a high-level programming language renowned for its clear syntax and readability, making it an ideal choice for both novice and experienced programmers. Developed by Guido van Rossum and first released in 1991, Python has evolved significantly over the years, becoming one of the most popular programming languages globally. Its versatility, coupled with extensive community support, has contributed to its widespread adoption across industries. As technology continues to advance, Python's role in developing applications that process and analyze complicated sets of data is expected to expand, securing its position as a leading tool for technological innovation in the years ahead.

## MICROSOFT EXCEL

Since its launch in 1985, Microsoft Excel has evolved from a basic spreadsheet tool into a comprehensive platform for data analysis, visualization, and automation. Originally intended for simple calculations, Excel has progressively incorporated advanced features such as pivot tables, intricate formulas, and Visual Basic for Applications (VBA), empowering users to automate tasks and develop custom functions. More recently, the integration of Python formulas in Excel represents a significant leap forward, enabling users to harness powerful data science tools and perform advanced analyses directly within Excel. This evolution underscores Excel's adaptability and its continued relevance in the dynamic, data-driven decision-making environment.

## RESEARCH OBJECTIVE

This article illustrates how ChatGPT can serve as a valuable digital assistant for medical librarians, offering:

(1) support in providing second opinions for their daily decisions,
(2) the ability to generate user-friendly Python scripts that automate repetitive tasks, particularly when processing and analyzing data collection such as Excel spreadsheets,
(3) tools to quickly summarize and synthesize relevant information from large volumes of data or literature, and
(4) the capability to translate or summarize information from languages in which the librarian may not be proficient.

The primary goal is to offer practical solutions that can significantly enhance the competence and effectiveness of medical librarians in their day-to-day responsibilities.

## INTEGRATED TOOL APPROACH

ChatGPT has emerged as a powerful tool for enhancing the productivity of information and data management by leveraging its capabilities with Excel spreadsheets and Python coding. By automating repetitive tasks, generating custom scripts, and providing real-time assistance in data processing, ChatGPT accelerates the construction, analysis, and synthesis of complex sets of information [5]. Its ability to seamlessly integrate with Excel allows users to manipulate and visualize data with ease, while its proficiency in Python enables the execution of advanced algorithms and data analysis techniques.

**Figure 1:**
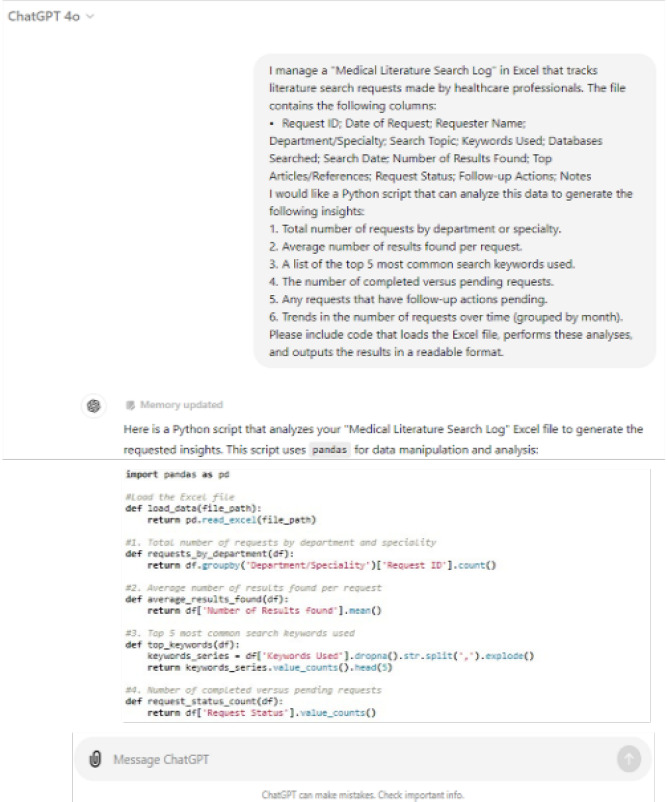
Using ChatGPT to create Python scripts

This combination not only streamlines workflows but also empowers users to derive insights more rapidly, making ChatGPT an invaluable asset in data-driven environments. For example, to have data entry automation, the combination of ChatGPT with Excel spreadsheets automates repetitive data entry tasks, and with Python programming generates scripts to automate data entry processes. This could be extended to a lot of other potential needs, such as data analysis, data visualization, formula assistance and data cleaning, integrating these possibilities as an automated analytical solution to manage and understand a database, as exemplified in Figure 1.

ChatGPT's ability to integrate with tools like Excel and Python has become indispensable in today's data-driven landscape. As the volume of information continues to grow, this capability empowers users to effectively organize and interpret sophisticated datasets while seamlessly integrating them [6]. By leveraging Excel for data organization and visualization, combined with Python's powerful coding features for advanced analysis and automation, ChatGPT facilitates faster and more accurate decision-making, enhancing productivity across various tasks.

## ANALYZING FROM A MEDICAL LIBRARIAN'S PERSPECTIVE

The adoption of ChatGPT and its ability to work with Excel and Python in the operational frameworks of medical libraries represents a pivotal enhancement in the methodologies for managing and analyzing information. ChatGPT is highly effective in assisting with data organization, analysis, and visualization in Excel, making it easier for librarians to handle elaborate data compilation and respond to user queries. Python, generated by ChatGPT, provides robust support for automating repetitive tasks and performing advanced data analysis, optimizing the storage, retrieval, and synthesis of information — that can be copy and pasted in Excel easily (Figure 1). Together, these tools create a comprehensive system that significantly improves the efficiency and effectiveness of library services, empowering medical librarians to manage the growing complexity of data in their daily work.

The integration of ChatGPT with Excel and Python offers a powerful boost to medical library operations (Table 2). With ChatGPT's natural language processing, medical librarians can easily manage queries and guide users through difficult data tasks. By utilizing Excel for data organization and Python for automation, they can streamline processes, improve accuracy, and enhance the support provided to healthcare professionals and researchers.

**Table 2:** Common use cases for using ChatGPT, Excel, and Python combined

Use Case	Role of ChatGPT	Role of Excel	Role of Python	Combined Impact
Information Retrieval	Helps formulate precise search queries and strategies	Organizes and retrieves data from extensive information set	Automates the extraction of data from various sources	Faster and more accurate retrieval of relevant information
User Interaction	Engages with users to clarify needs and provide tailored responses	Displays and organizes user-requested data in a clear format	Automates user queries and data processing tasks	Enhanced user experience through efficient, responsive interactions
Data Management	Assists in structuring and categorizing large sets of data	Manages data organization using tables, filters, and pivot tables	Automates data management processes, including cleaning and integration	Streamlined data management with reduced manual effort and increased accuracy.
Research Assistance	Provides guidance on research methodologies and data analysis	Facilitates data analysis and presentation through charts and reports	Executes advanced data analyses and simulations	Comprehensive support for research, leading to deeper insights and quicker results
Educational Support	Offers instructional content and explanations for data-related tasks	Organizes educational materials and examples in a structured format	Automates the generation of educational data repository and simulations	Enhanced learning experience with interactive, datadriven resources

However, adopting ChatGPT, Excel, and Python also comes with challenges. These include a learning curve, adapting workflows, and ensuring smooth integration with existing systems. Data security and privacy are additional concerns that require careful attention. Addressing these issues through proper training, infrastructure investment, and proactive management is key to unlocking the full potential of these tools and improving library services.

## CONCLUSION

This article demonstrates how the combination of ChatGPT, Excel, and Python can greatly enhance the productivity of medical librarians by automating repetitive tasks and improving data management processes. By utilizing ChatGPT's ability to interact with Excel spreadsheets and generate Python code, medical librarians can competently analyze and handle large datasets, streamlining their daily operations. The exploration of these technologies underscores their transformative potential in boosting effectiveness, accuracy, and the personalization of library services. However, successful integration requires careful planning and management. Looking ahead, ongoing advancements in AI and programming promise even greater improvements in productivity, enabling librarians to take on more strategic roles supported by technology.
